# Supplementary material for: COVID-19 Pandemic and the Crisis of Health Systems: The Experience of the Apulia Cancer Network and of the Comprehensive Cancer Center Istituto Tumori “Giovanni Paolo II” of Bari
Source: Int J Environ Res Public Health. 2020 Apr 16;17(8):2763. doi: 10.3390/ijerph17082763 (PMC7216232; doi:10.3390/ijerph17082763)
Supplement: Supplementary file 1 [file ijerph-17-02763-s001.pdf]

IRCCS ISTITUTO TUMORI "GIOVANNI PAOLO II"

Sede Legale: Via Orazio Flacco, 65 - 70124 Bari

www.oncologico.bari.it

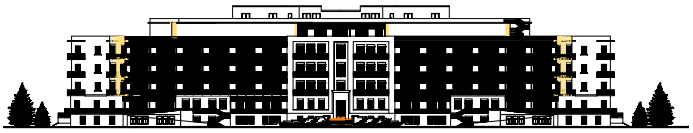

DOCUMENTO DI VALUTAZIONE DEI RISCHI

Ex art. 28 d.lgs. 9 aprile 2008, n° 81

Documento di Valutazione dei Rischi

CORONA VIRUS CoVID-19

Versione 03

Approvato il 20 MARZO 2020

Dai componenti del SPP

**Datore di Lavoro/Direttore Generale**	Dott. Antonio Delvino
**RSPP**	ing. Giancarlo Salomone
**Medico Competente**	d.ssa Ilaria Tatò
**RLS**	dott. Domenico Rosario Losacco
**RLS**	dott. Giovanni Izzi
**RLS**	dott.ssa Stefania Tommasi

Per la particolare rilevanza dell'argomento trattato, hanno partecipato alla riunione del SPP e approvano il presente documento anche:

**Direttore Sanitario**	dott. Pietro Milella
